# En bloc stapler versus separate clip ligation for renal hilar control during robotic‐assisted nephrectomy: A retrospective comparative cohort study

**DOI:** 10.1002/bco2.70297

**Published:** 2026-10-16

**Authors:** Ibrahim A. Khalil, Fatima A. Al‐Jassim, Yaser M. Ata, Humam Emad Rajha, Abdulhamed Mansour, Ibrahim Al‐Emadi, Khalid Al‐Jalham, Khalid Al Rumaihi

**Affiliations:** ^1^ Department of Urology Hamad Medical Corporation Doha Qatar; ^2^ College of Medicine, QU Health Qatar University Doha Qatar; ^3^ Department of Urology, College of Medicine Qatar University Doha Qatar; ^4^ Department of Clinical Urology Weill Cornell Medicine‐Qatar Doha Qatar

**Keywords:** en bloc ligation, Hem‐o‐Lok clips, robotic nephrectomy, vascular control

## Abstract

**Objectives:**

This study aimed to compare en bloc endovascular stapler ligation (ENBLOC) with separate clip ligation (CLIPS) for renal hilar control during robotic‐assisted nephrectomy, with emphasis on perioperative safety.

**Patients and methods:**

This single‐centre retrospective cohort included 193 consecutive adults undergoing robotic‐assisted radical nephrectomy, simple nephrectomy or nephroureterectomy between January 2018 and December 2025. Patients were classified as CLIPS (*n* = 66) or ENBLOC (*n* = 127). Primary safety outcomes were intraoperative complications, postoperative complications and postoperative arteriovenous fistula (AVF) formation. Operative, renal functional and oncological outcomes were secondary or exploratory. Procedure‐specific subgroup analyses were also performed.

**Results:**

Baseline characteristics were largely comparable, except for ASA III–IV status was more frequent in the CLIPS group (27.3% vs. 16.5%, *p* = 0.029). Operative time (206.5 vs. 218.0 min, *p* = 0.26), estimated blood loss (median 100 mL in both groups, *p* = 0.37) and transfusion rates (1.5% vs. 5.5%, *p* = 0.19) did not differ significantly. Intraoperative complications occurred in 3/66 (4.5%) CLIPS and 8/127 (6.3%) ENBLOC patients (OR 1.41, 95% CI 0.36–5.51; absolute risk difference 1.8 percentage points, 95% CI − 4.8 to 8.3; *p* = 0.752). Postoperative complications occurred in 5/66 (7.6%) and 9/127 (7.1%) patients, respectively (*p* = 0.91). No AVF was identified during follow‐up. Exploratory subgroup analyses did not identify additional statistically significant between‐group differences, although estimates were limited by small sample sizes and sparse events.

**Conclusion:**

En bloc stapler ligation was not associated with statistically significant differences in the evaluated safety outcomes compared with separate clip ligation. However, the observational design and limited number of events preclude conclusions regarding equivalence, and clinically relevant differences cannot be excluded.

## 1 INTRODUCTION

Nephrectomy and nephroureterectomy are among the most commonly performed urological procedures, with minimally invasive laparoscopic and robotic approaches widely adopted for benign and malignant renal disease.^1^, ^2^, ^3^, ^4^, ^5^ Since the introduction of laparoscopic nephrectomy, minimally invasive surgery has demonstrated oncologic outcomes comparable to open surgery while reducing perioperative morbidity.^6^, ^7^, ^8^ The robotic platform has further enhanced surgical precision, visualization and ergonomics, facilitating increasingly complex renal procedures.^9^, ^10^

A critical step in these operations is secure control of the renal hilum. Traditionally, this is achieved through individual ligation of the renal artery and vein using polymer locking clips.^1^, ^11^, ^12^ However, this approach may be technically challenging because of limited working space and the posterior anatomical position of the renal artery relative to the vein.^13^ Hilar dissection may also increase operative complexity and the risk of vascular injury, particularly in patients with inflammation, adhesions or distorted anatomy.^1^, ^12^

En bloc ligation of the renal hilum, in which the renal artery and vein are controlled simultaneously using an endovascular stapler, has therefore been proposed as an alternative technique. Potential advantages include simplified hilar dissection, rapid vascular control and reduced technical difficulty in complex cases.^14^, ^15^, ^16^, ^17^, ^18^ Previous studies have reported acceptable perioperative outcomes with en bloc stapling and, in some series, shorter operative time without increased transfusion or complication rates compared with separate vessel ligation.^1^, ^2^, ^11^, ^12^

Despite these potential advantages, concerns regarding en bloc hilar control have historically included arteriovenous fistula (AVF) formation, thought to result from the close proximity of arterial and venous stumps after simultaneous ligation.^19^, ^20^, ^21^, ^22^, ^23^ as well as stapler malfunction or incomplete vascular control.^24^, ^25^ More contemporary studies using modern stapling devices have reported few or no postoperative AVFs, including across relatively large cohorts and extended follow‐up, although this rare complication remains clinically relevant.^1^, ^11^, ^26^, ^27^, ^28^, ^29^, ^30^ Separate clip ligation is also not without limitations and may require more extensive hilar dissection in anatomically or technically challenging cases.^12^, ^31^

Most comparative evidence regarding these vascular control strategies has been derived from conventional laparoscopic nephrectomy. However, robotic surgery provides three‐dimensional visualization, articulated instrumentation and greater dexterity, which may facilitate individual renal vessel dissection and potentially reduce some of the technical advantages previously attributed to en bloc stapling. Therefore, findings from laparoscopic surgery may not necessarily be directly extrapolated to the robotic setting. Although robotic‐assisted nephrectomy has demonstrated perioperative outcomes comparable to laparoscopic approaches,^32^, ^33^ comparative evidence specifically addressing renal hilar control techniques during robotic surgery remains limited.

Given this evidence gap, the present study aimed to compare en bloc stapler ligation (ENBLOC) with separate clip ligation (CLIPS) for renal hilar control during robotic‐assisted nephrectomy, with primary emphasis on perioperative safety and secondary assessment of operative, functional and exploratory oncological outcomes.

## 2 METHODS

### 2.1 Study design and patient population

This single‐centre retrospective cohort study included consecutive adult patients (≥18 years) who underwent robotic‐assisted radical nephrectomy, nephroureterectomy or simple nephrectomy for benign or malignant indications between January 2018 and December 2025 at a high‐volume tertiary care centre. Patients with incomplete perioperative or follow‐up data or those undergoing nonrobotic surgery were excluded. The study was conducted following institutional review board approval.

### 2.2 Surgical technique and group allocation

All procedures were performed by experienced robotic surgeons using the da Vinci Xi Surgical System (Intuitive Surgical, Sunnyvale, CA, USA). Hilar‐control technique was selected by the operating surgeon according to intraoperative assessment of hilar anatomy and technical feasibility rather than a standardized institutional protocol. Detailed hilar anatomy variables, including vascular number and branching, anomalous vasculature, hilar adenopathy, adhesions and inflammatory changes, were not systematically captured and therefore could not be formally compared between groups. Surgeon‐specific technique preference was also not available as a structured variable. Potential temporal changes in practice were assessed by examining technique use according to calendar year.

Patients were classified according to the primary method of renal pedicle control:

- Separate clip ligation (CLIPS): The renal artery and vein were individually dissected and sequentially ligated using large (L, purple) nonabsorbable Hem‐o‐Lok polymer clips, with two clips placed on the patient side and one on the specimen side before vessel division.
- En bloc stapler ligation (ENBLOC): The principal renal hilar pedicle was controlled simultaneously using an endovascular stapler (Endo GIA, Covidien/Medtronic) without routine separation of the renal artery and vein. A 35‐ or 45‐mm stapler cartridge was selected according to hilar anatomy and technical requirements.

### 2.3 Data collection

Demographic, preoperative, operative, pathological, postoperative and follow‐up data were obtained from electronic medical records, operative reports, pathology reports and imaging studies. Collected variables included age, sex, body mass index, comorbidities, American Society of Anesthesiologists (ASA) score, procedure type, operative side, operative time, estimated blood loss, transfusion requirement and perioperative complications. Operative time was defined from skin incision to completion of skin closure.

Postoperative renal function was assessed descriptively using serum creatinine and estimated glomerular filtration rate (eGFR; CKD‐EPI equation) at postoperative Days 1 and 30 and the last available follow‐up. Postoperative complications were classified according to the Clavien–Dindo system,^34^ with Grades I–II considered minor and Grade ≥III considered major complications. Length of hospital stay, 30‐day readmission and duration of follow‐up were also recorded.

Among patients with malignant pathology, histological subtype, surgical margin status and radiological recurrence were evaluated descriptively as exploratory oncological outcomes. Recurrence status was based on radiological findings obtained during routine postoperative surveillance. Because recurrence patterns differ between renal cell carcinoma (RCC) and upper tract urothelial carcinoma, recurrence findings were interpreted descriptively according to tumour type. Surveillance followed routine clinical practice rather than a study‐specific protocol, and exact recurrence‐event dates were not consistently available for time‐to‐event analysis.

AVF occurrence was assessed retrospectively using postoperative contrast‐enhanced imaging and clinical documentation. All patients underwent contrast‐enhanced imaging during routine postoperative follow‐up; however, imaging timing and protocols were not standardized specifically for AVF surveillance, and no dedicated AVF‐specific protocol was used.

### 2.4 Outcomes

The primary safety outcomes were intraoperative complications, postoperative complications and postoperative AVF formation. For interpretive clarity, these outcomes were designated as primary safety outcomes for the present retrospective analysis and were not prospectively prespecified.

Secondary and exploratory outcomes included operative duration, estimated blood loss, transfusion requirement, hospital stay, 30‐day readmission, renal functional parameters and oncological findings among patients with malignant disease.

### 2.5 Statistical analysis

Statistical analyses were performed using Stata SE version 19.5 (StataCorp LLC, College Station, TX, USA). Continuous variables were assessed for normality using histograms and density plots. Normally distributed variables were summarized as mean ± standard deviation and compared using independent two‐sample *t*‐tests, while non‐normally distributed variables were reported as median with interquartile range and compared using Wilcoxon rank‐sum tests. Categorical variables were reported as frequencies and percentages and compared using Pearson's chi‐squared test or Fisher's exact test, as appropriate. All tests were two‐sided, with *p* < 0.05 considered statistically significant.

For the primary safety outcomes, between‐group comparisons were performed using appropriate categorical tests. Odds ratios and absolute risk differences with 95% confidence intervals were reported for intraoperative complications, and corresponding absolute estimates were reported for postoperative complications where applicable. AVF formation was summarized descriptively because no events occurred in either group.

Because all eligible consecutive patients during the predefined study period were included, no a priori sample‐size calculation was performed. A sensitivity power analysis based on the intraoperative complication outcome was performed to characterize the magnitude of between‐group differences detectable with the available sample. No equivalence or noninferiority margin was prespecified, and the analyses were therefore not interpreted as demonstrating equivalence or noninferiority.

Given the limited number of intraoperative complication events, extensive multivariable modelling was avoided to reduce the risk of overfitting. Exploratory sensitivity analyses were performed within the radical nephrectomy cohort using separate parsimonious logistic regression models adjusting individually for ASA III–IV status, chronic kidney disease, tumour size and calendar year of surgery. These analyses were intended to assess the robustness of the unadjusted association rather than provide definitive control of confounding.

Renal function analyses were considered descriptive because a structured baseline renal function measurement was unavailable in the analytical dataset. Radical nephrectomy represented the principal procedure‐specific subgroup, while analyses of simple nephrectomy and nephroureterectomy were considered exploratory because of their smaller sample sizes. Formal interaction testing according to procedure type was not performed because of limited subgroup sizes and sparse outcome events.

## 3 RESULTS

### 3.1 Overall cohort and baseline characteristics

A total of 193 consecutive patients who underwent robotic‐assisted nephrectomy were included in the analysis, comprising 66 patients (34.2%) in the CLIPS group and 127 patients (65.8%) in the ENBLOC group. Baseline demographic and clinical characteristics are presented in Table 1. The two groups were well balanced with respect to age (50.3 vs. 51.1 years, *p* = 0.70), sex distribution (69.7% vs. 73.2% male, *p* = 0.60), body mass index (28.3 vs. 28.5 kg/m^2^, *p* = 0.87) and the prevalence of major comorbidities, including diabetes mellitus (37.9% vs. 26.0%, *p* = 0.087), hypertension (66.7% vs. 63.0%, *p* = 0.59), coronary artery disease (10.6% vs. 5.5%, *p* = 0.16) and chronic kidney disease (21.2% vs. 11.0%, *p* = 0.057). Patients in the ENBLOC group had a lower prevalence of ASA III–IV classification than those in the CLIPS group (16.5% vs. 27.3%, *P* = 0.029). The distribution of surgical procedures is presented in Table 1 and illustrated in Figure 1.

**TABLE 1:** Baseline patient characteristics.

Variable	Clips (*n* = 66)	ENBLOC (*n* = 127)	*P‐*value
Age, years, mean (SD)	50.3 (13.2)	51.1 (12.8)	0.70
Male sex, *n* (%)	46 (69.7)	93 (73.2)	0.60
BMI, kg/m^2^, mean (SD)	28.3 (5.2)	28.5 (5.1)	0.87
Diabetes mellitus, *n* (%)	25 (37.9)	33 (26.0)	0.087
Hypertension, *n* (%)	44 (66.7)	80 (63.0)	0.59
Chronic kidney disease, *n* (%)	14 (21.2)	14 (11.0)	0.057
Coronary artery disease, *n* (%)	7 (10.6)	7 (5.5)	0.16
ASA III–IV, *n* (%)	18 (27.3)	21 (16.5)	0.029
Procedure type, *n* (%)	—	—	<0.001
Radical nephrectomy	38 (57.6)	105 (82.7)	—
Simple nephrectomy	18 (27.3)	11 (8.7)	—
Nephroureterectomy	10 (15.2)	11 (8.7)	—
Follow‐up, months, median (IQR)	17 (10–28)	19 (12–32)	0.64

**FIGURE 1:**
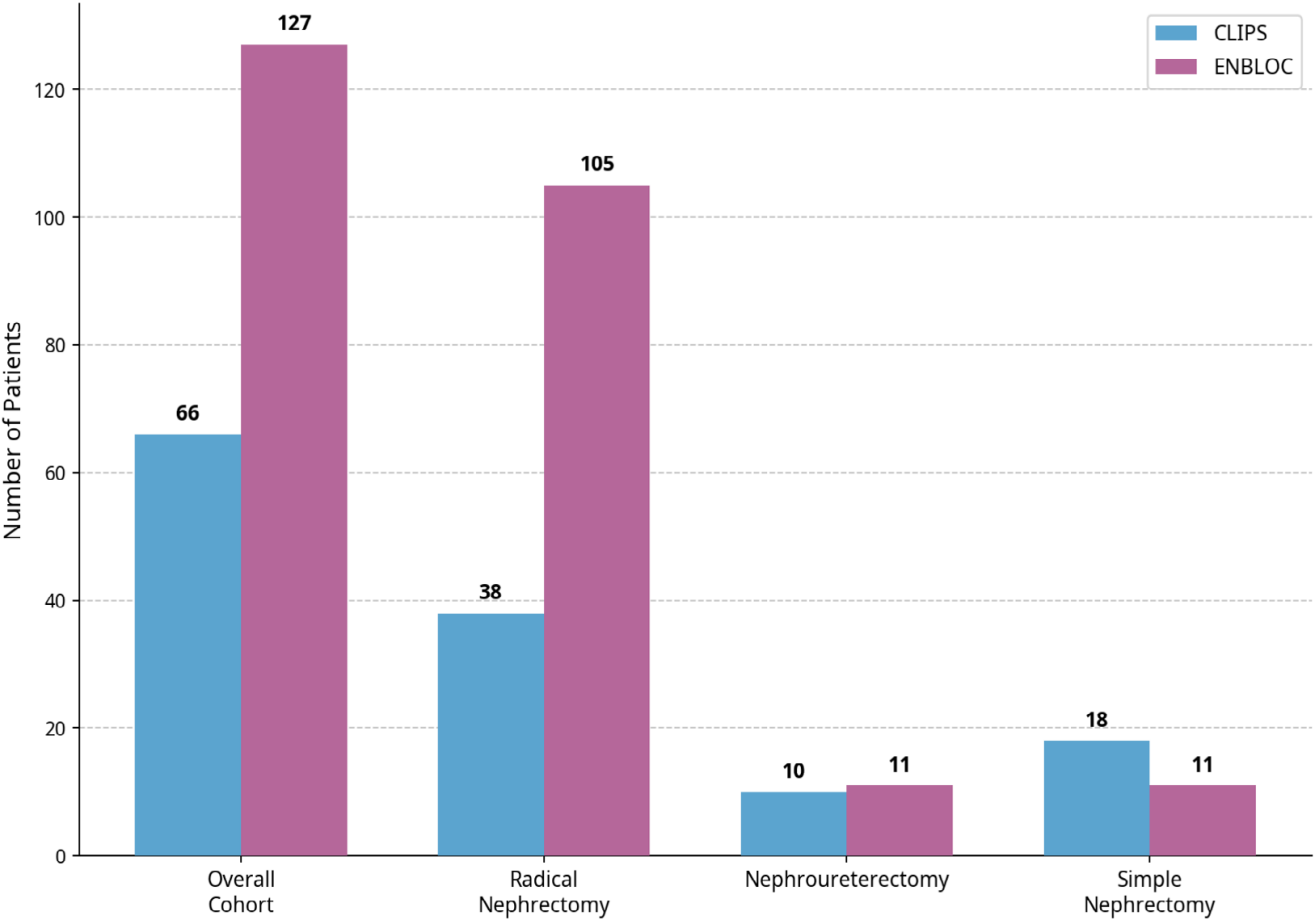
Patient distribution by surgery type.

The distribution of hilar‐control techniques varied over the study period. CLIPS predominated during the early years of the cohort, whereas ENBLOC became more frequently used from 2021 onward. Nevertheless, both techniques continued to be used contemporaneously between 2021 and 2025, indicating that ENBLOC did not completely replace CLIPS during the study period. The annual number of procedures by hilar‐control technique is shown in Figure 2.

**FIGURE 2:**
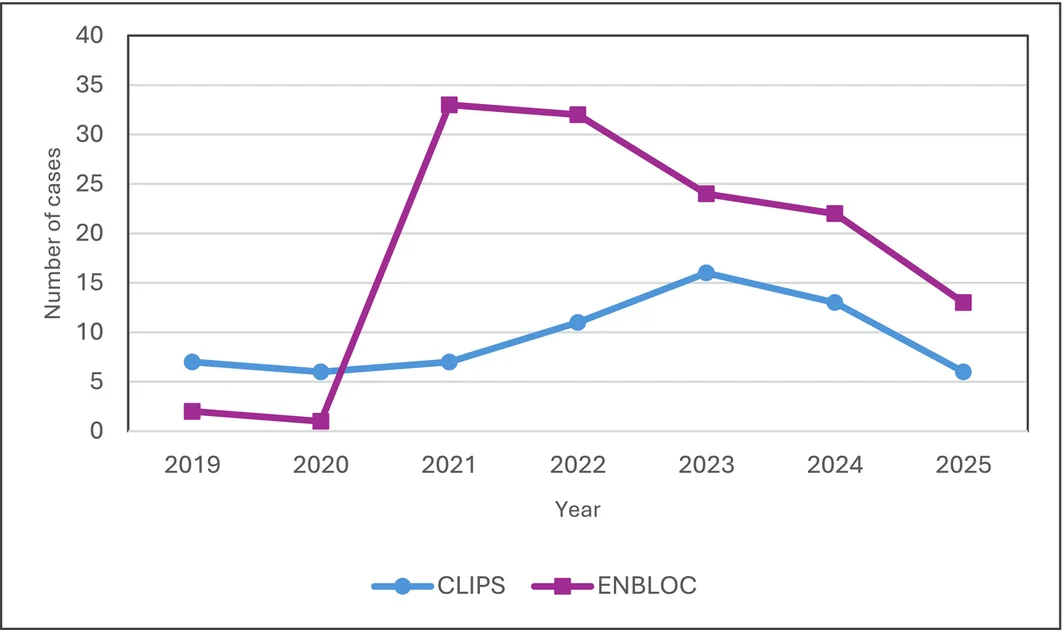
Annual number of procedures by hilar‐control technique (CLIPS vs. ENBLOC).

### 3.2 Perioperative outcomes

Perioperative outcomes are summarized in Tables 2 and 3 and illustrated in Figures 3 and 4. Operative duration did not differ significantly between the CLIPS and ENBLOC groups (206.5 vs. 218.0 min, *p* = 0.26) nor did estimated blood loss (median 100 mL in both groups, *p* = 0.37) or transfusion rates (1.5% vs. 5.5%, *p* = 0.19). Intraoperative complications occurred in 3/66 (4.5%) CLIPS and 8/127 (6.3%) ENBLOC patients, corresponding to an absolute risk difference of 1.8 percentage points (95% CI, −4.8 to 8.3) and an OR of 1.41 (95% CI, 0.36–5.51; Fisher's exact *p* = 0.752). Postoperative complications occurred in 5/66 (7.6%) CLIPS and 9/127 (7.1%) ENBLOC patients (*p* = 0.91). Clavien–Dindo Grades I and II complications occurred in 5 (7.6%) and 8 (6.3%) patients, respectively, while one Grade IVa complication occurred in the ENBLOC group. Hospital stay (3.8 vs. 4.2 days, *p* = 0.70) and 30‐day readmission (0% vs. 1.6%, *p* = 0.30) did not differ significantly. No endovascular stapler malfunction was documented.

**TABLE 2:** Operative and tumour characteristics.

Variable	Clips (*n* = 66)	ENBLOC (*n* = 127)	(95% CI)	*P*‐value
Operative time, min, mean (SD)	206.5 (81.3)	218.0 (94.2)	+11.5 min (−14.3 to 37.3)	0.26
Estimated blood loss, mL, median (IQR)	100 (50–150)	100 (50–200)	—	0.37
Blood transfusion, *n* (%)	1 (1.5)	7 (5.5)	+4.0% (−0.9 to 8.9)	0.19
Tumour size, cm, mean (SD)	6.8 (3.9)	6.9 (4.1)	+0.1 cm (−1.1 to 1.3)	0.82
Malignant pathology, *n* (%)	45 (68.2)	106 (83.5)	—	0.015
Benign pathology, *n* (%)	21 (31.8)	21 (16.5)	—	—
Negative surgical margin, *n* (%)	61 (92.4)	122 (96.1)	—	0.27

**TABLE 3:** Perioperative complications and recovery.

Variable	Clips (*n* = 66)	ENBLOC (*n* = 127)	(95% CI)	*P*‐value
Intraoperative complications, *n* (%)	3 (4.5)	8 (6.3)	+1.8% (−4.8 to 8.3)	0.752^a^
Postoperative complications, *n* (%)	5 (7.6)	9 (7.1)	−0.5% (−8.3 to 7.3)	0.91
Clavien–Dindo grade I–II, *n* (%)	5 (7.6)	8 (6.3)	—	—
Clavien–Dindo grade ≥ III, *n* (%)	0 (0)	1 (0.8)	—	—
Arteriovenous fistula formation, *n* (%)	0	0	—	—
Hospital stay, days, mean (SD)	3.8 (4.2)	4.2 (8.7)	+0.4 days (−1.4 to 2.2)	0.70
30‐day readmission, *n* (%)	0	2 (1.6)	—	0.30

^a^ Fisher's exact test.

**FIGURE 3:**
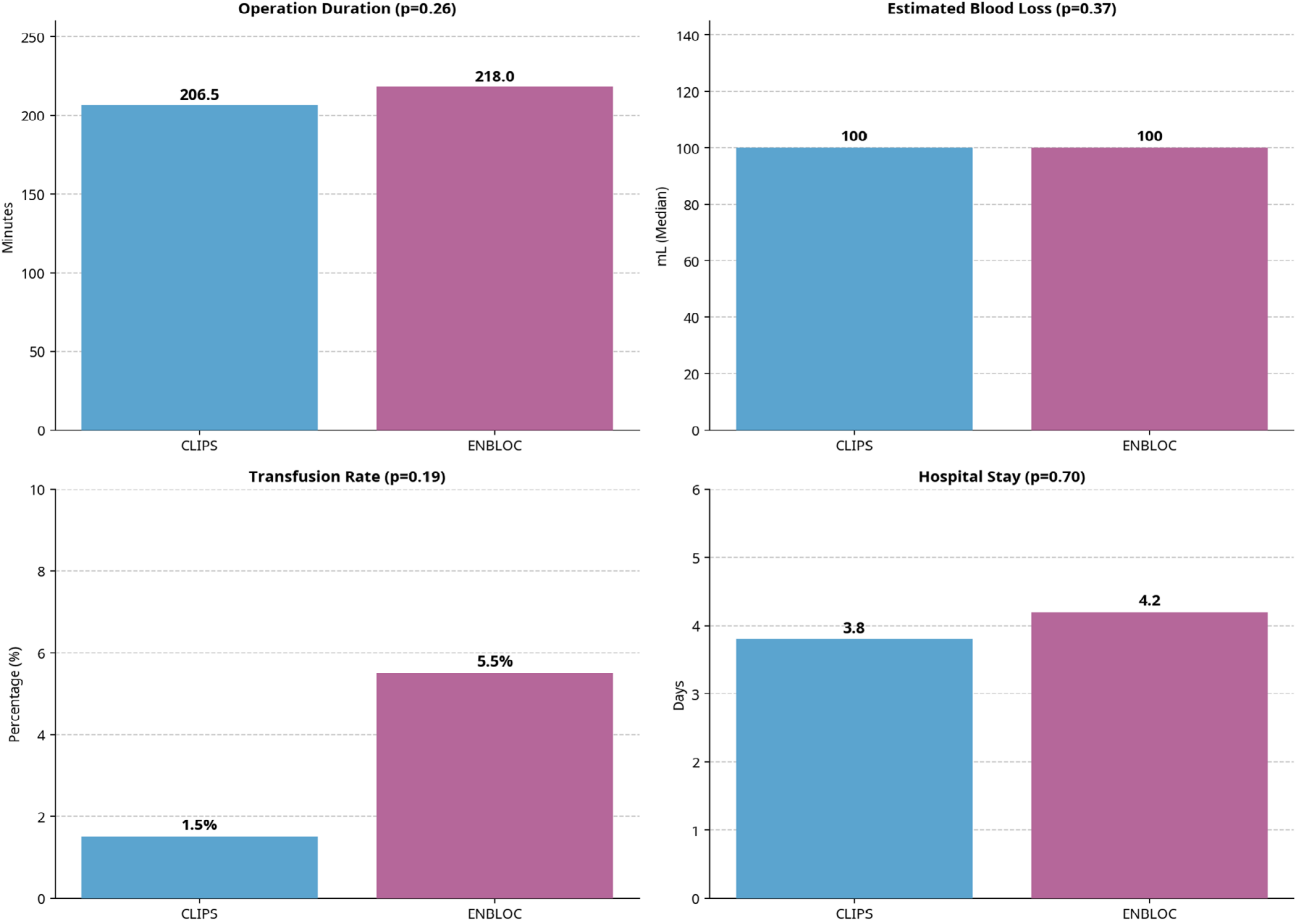
Perioperative outcomes: CLIPS versus ENBLOC. Comparison of (A) operation duration, (B) estimated blood loss, (C) transfusion rate and (D) hospital stay. No statistically significant differences were observed between CLIPS and ENBLOC groups.

**FIGURE 4:**
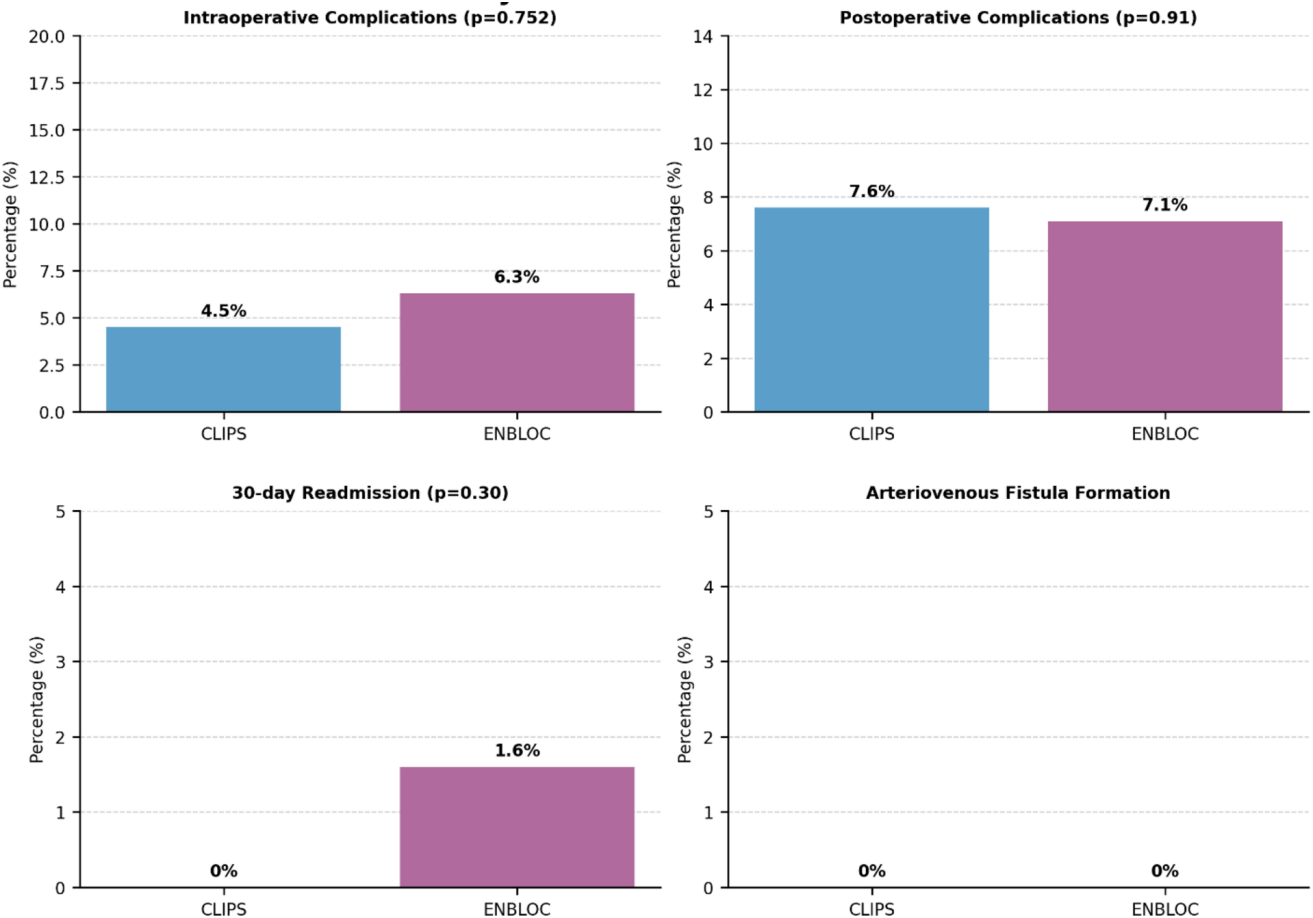
Safety outcomes: CLIPS versus ENBLOC. Comparison of (A) intraoperative complications, (B) postoperative complications, (C) 30‐day readmission and (D) arteriovenous fistula (AVF) formation. No statistically significant differences were observed. No AVF was identified in either group during the available follow‐up.

### 3.3 Postoperative renal function

Postoperative renal function parameters are presented descriptively in Table 4 and Figure 5. No statistically significant between‐group differences were identified in serum creatinine or eGFR at postoperative Days 1 and 30 or the last available follow‐up. Serum creatinine was 128.5 vs. 119.3 μmol/L at Day 1 (*p* = 0.18), 129.1 vs. 135.7 μmol/L at Day 30 (*p* = 0.91) and 122.0 vs. 131.3 μmol/L at last follow‐up (*p* = 0.88) for CLIPS and ENBLOC, respectively. Corresponding eGFR values were 71.6 vs. 71.1 mL/min/1.73 m^2^ (*p* = 0.15), 60.3 vs. 60.2 mL/min/1.73 m^2^ (*p* = 0.67) and 60.7 vs. 61.0 mL/min/1.73 m^2^ (*p* = 0.35). These analyses were descriptive and were not adjusted for baseline renal function.

**TABLE 4:** Postoperative haematological and renal function parameters.

Variable	Clips (*n* = 66)	ENBLOC (*n* = 127)	*P*‐value
Preoperative haemoglobin, g/dL	13.1 (1.8)	13.2 (1.9)	0.84
Postoperative haemoglobin, g/dL	12.3 (1.5)	12.3 (1.8)	0.95
POD1 haemoglobin, g/dL	12.0 (1.7)	12.0 (1.9)	1.00
POD1 creatinine, μmol/L	128.5 (157.9)	119.3 (91.5)	0.18
POD30 creatinine, μmol/L	129.1 (90.2)	135.7 (136.1)	0.91
Last follow‐up creatinine, μmol/L	122.0 (93.3)	131.3 (135.0)	0.88
POD1 eGFR, mL/min/1.73 m^2^	71.6 (22.1)	71.1 (21.8)	0.15
POD30 eGFR, mL/min/1.73 m^2^	60.3 (18.9)	60.2 (18.5)	0.67
Last follow‐up eGFR, mL/min/1.73 m^2^	60.7 (20.6)	61.0 (22.0)	0.35

**FIGURE 5:**
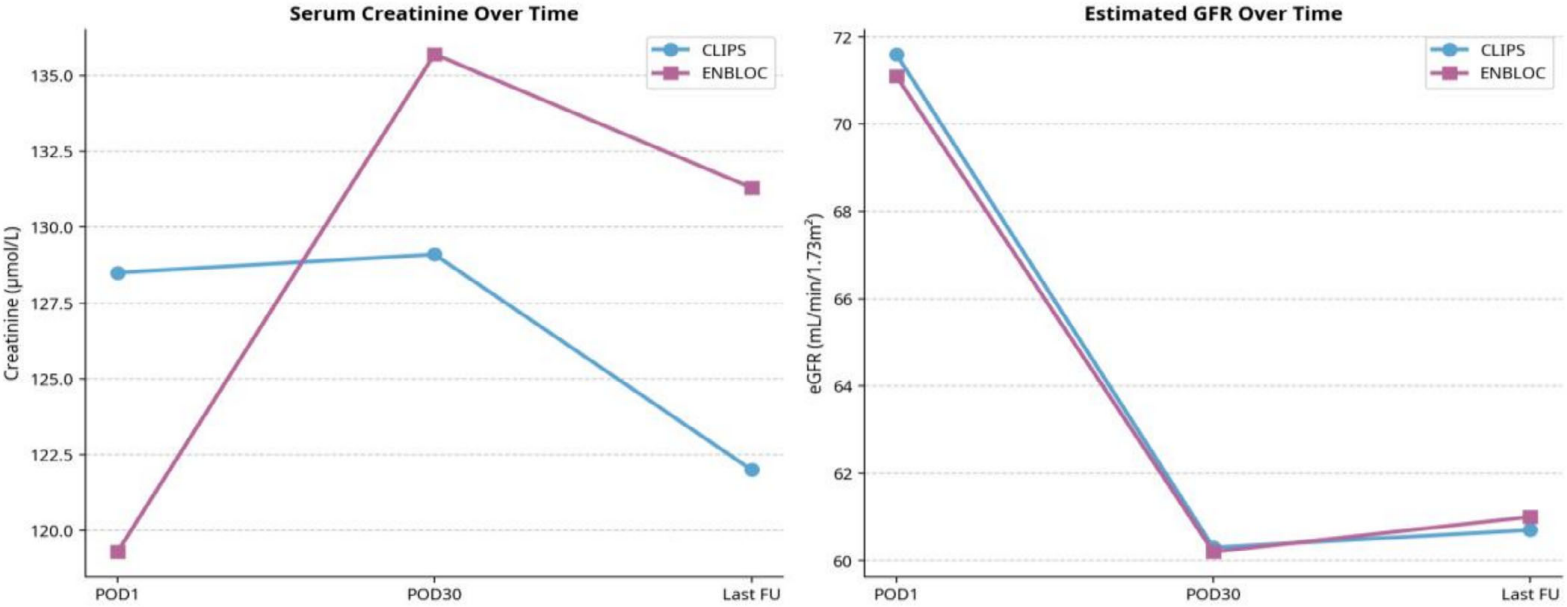
Postoperative renal function: CLIPS versus ENBLOC. Descriptive comparison of (A) serum creatinine and (B) estimated glomerular filtration rate (eGFR) at postoperative Days 1 and 30 and last available follow‐up. No statistically significant between‐group differences were observed at the evaluated time points. These comparisons were descriptive and were not adjusted for baseline renal function.

### 3.4 AVF during follow‐up

All patients underwent postoperative contrast‐enhanced imaging during follow‐up. No AVF was identified in either group. Because imaging timing and protocols were not standardized specifically for AVF surveillance and AVF is a rare potentially delayed complication, this finding was interpreted descriptively.

### 3.5 Pathological and oncological findings

Pathological and oncological findings among patients with malignant disease were considered exploratory. Malignant pathology was present in 45/66 (68.2%) CLIPS and 106/127 (83.5%) ENBLOC patients (*p* = 0.015). Histological subclassification is presented in supplementary Table S1, with clear‐cell RCC representing the predominant malignant histology in both groups. At the last available follow‐up, recurrence was identified in 7/45 (15.6%) CLIPS and 17/106 (16.0%) ENBLOC patients. Among patients with RCC, recurrence occurred in 7/43 (16.3%) CLIPS and 16/100 (16.0%) ENBLOC patients, whereas among patients with upper tract urothelial carcinoma, recurrence occurred in 0/2 and 1/5, respectively. These findings were interpreted descriptively because of heterogeneous tumour types, limited disease‐specific subgroup sizes and nonstandardized oncological follow‐up.

### 3.6 Principal procedure‐specific analysis: Robotic radical nephrectomy

Robotic radical nephrectomy was the largest procedure‐specific subgroup (*n* = 143), comprising 38 CLIPS and 105 ENBLOC patients. The ENBLOC group had a lower prevalence of ASA III–IV status (*p* = 0.013) and a longer median follow‐up duration (19 vs. 13.5 months, *p* = 0.035). Tumour size was numerically larger in the ENBLOC group (7.25 vs. 6.41 cm, *p* = 0.093). No additional statistically significant between‐group differences were identified in the evaluated perioperative outcomes. All intraoperative complications in the overall cohort occurred within this subgroup: 3/38 (7.9%) in CLIPS and 8/105 (7.6%) in ENBLOC (unadjusted OR 0.96, 95% CI 0.24–3.83). In exploratory sensitivity analyses, the estimated association remained materially unchanged after separate adjustment for ASA III–IV status (OR 1.28, 95% CI 0.30–5.52), chronic kidney disease (OR 0.92, 95% CI 0.23–3.71), tumour size (OR 0.84, 95% CI 0.20–3.42) and calendar year (OR 1.01, 95% CI 0.25–4.08). Confidence intervals remained wide, reflecting the limited number of events.

### 3.7 Exploratory subgroup: Robotic simple nephrectomy

A total of 29 patients underwent robotic simple nephrectomy (18 CLIPS and 11 ENBLOC). Diabetes mellitus was more frequent in the CLIPS group, although the difference did not reach statistical significance (28% vs. 0%, *p* = 0.055). No additional statistically significant between‐group differences were identified in the evaluated outcomes, and no complications or AVFs were observed. Given the small subgroup size, these findings were considered exploratory.

### 3.8 Exploratory subgroup: Robotic nephroureterectomy

A total of 21 patients underwent robotic nephroureterectomy (10 CLIPS and 11 ENBLOC). Left‐sided procedures were more frequent in the CLIPS group (90% vs. 27%, *p* = 0.004). No additional statistically significant between‐group differences were identified in the evaluated outcomes. Given the small subgroup size, these findings were considered exploratory and should not be interpreted as evidence of equivalent outcomes.

## 4 DISCUSSION

Our findings are consistent with previous laparoscopic studies demonstrating that en bloc ligation using vascular staplers is an efficient and safe technique, often associated with reduced operative time without an increase in perioperative complications or transfusion requirements.^10^, ^11^, ^13^, ^14^, ^15^ The theoretical advantage of en bloc ligation lies in simplifying hilar dissection, particularly in patients with complex anatomy, inflammation or adhesions, where isolation of individual renal vessels may be technically challenging.^16^, ^17^ In our cohort, however, operative time did not differ significantly between the two techniques. This finding may reflect surgeon experience and the technical advantages of the robotic platform, which facilitate precise hilar dissection and may attenuate the operative time differences reported in earlier laparoscopic studies.

An important consideration in interpreting these findings is the anatomical complexity of the renal hilum, which may influence both technical feasibility and the surgeon's choice of vascular control strategy. Factors such as accessory renal vessels, early arterial branching, anomalous vascular anatomy, bulky hilar disease, inflammatory changes, and adhesions may affect the practicality of en bloc stapling versus separate vessel ligation. In the present retrospective cohort, these anatomical features were not systematically captured in a structured manner and therefore could not be compared between groups or incorporated into the analysis. Consequently, the possibility that patients with more complex hilar anatomy were preferentially managed using one technique cannot be excluded and represents a potential source of confounding by indication. The importance of hilar anatomy and operative complexity in robotic renal surgery has also been highlighted in dedicated series of technically challenging hilar tumours, including the experience reported by Ferriero et al.,^35^ although that study evaluated robot‐assisted partial nephrectomy rather than extirpative nephrectomy. These considerations underscore the need to interpret the present findings within the context of individualized intraoperative anatomical assessment.

Technique utilization also changed over the study period, with ENBLOC becoming more frequently used from 2021 onward, while both approaches remained in concurrent use through 2025. Although calendar year was examined in exploratory sensitivity analyses, residual confounding related to evolving surgeon preference, cumulative robotic experience and temporal changes in case selection cannot be excluded.

A key historical concern regarding en bloc hilar ligation is the potential development of AVF, attributed to the close approximation of arterial and venous stumps following simultaneous ligation.^18^, ^19^, ^20^, ^21^ Early reports suggested a possible association with clinically significant sequelae, including high‐output cardiac failure.^19^, ^21^, ^22^ However, more contemporary evidence has challenged this paradigm, with multiple studies reporting no cases of AVF following en bloc ligation using modern stapling devices.^10^, ^25^, ^26^, ^27^, ^28^, ^29^ No AVF was identified in either group during the available follow‐up, and all patients underwent postoperative contrast‐enhanced imaging. This finding is reassuring; however, it should be interpreted cautiously because AVF is a rare complication that may present after prolonged latency, and imaging was performed as part of routine clinical surveillance rather than according to a dedicated AVF‐specific protocol. Accordingly, the absence of observed AVF in this cohort does not exclude a small risk of rare or delayed AVF formation following en bloc hilar ligation.

Intraoperative complications were uncommon in both groups, with an absolute observed difference of approximately 1.8%. However, the estimate was imprecise (OR 1.41, 95% CI 0.36–5.51), and the study therefore cannot exclude clinically relevant differences in complication risk between the techniques. Accordingly, the absence of statistical significance should not be interpreted as demonstrating equivalence or noninferiority. The limited number of events and wide confidence intervals indicate that clinically relevant differences between techniques cannot be excluded.

The inclusion of radical nephrectomy, simple nephrectomy and nephroureterectomy introduces clinical heterogeneity because these procedures differ in indication, inflammatory burden, operative complexity, postoperative recovery and oncological context. For this reason, the radical nephrectomy cohort, which represented the majority of the study population, was emphasized as the principal procedure‐specific comparative population. The simple nephrectomy and nephroureterectomy analyses should be regarded as exploratory because of their limited sample sizes. The pooled analysis therefore reflects the overall technical experience with hilar‐control strategies across robotic extirpative renal surgery rather than a procedure‐specific estimate of effect.

Renal functional findings in the present study were secondary and should be interpreted descriptively. Following radical nephrectomy, postoperative renal function is influenced by baseline renal reserve and patient‐related factors, including age, diabetes, hypertension, proteinuria, and preoperative eGFR, as well as compensatory function of the contralateral kidney.^36^, ^37^ In the present cohort, postoperative creatinine and eGFR did not demonstrate statistically significant between‐group differences at the evaluated time points; however, structured baseline renal function measurements suitable for change‐from‐baseline or longitudinal modelling were unavailable. Accordingly, these findings should not be interpreted as evidence of equivalent renal functional preservation between CLIPS and ENBLOC.

Oncological findings were likewise exploratory. Recurrence after nephrectomy for RCC is primarily determined by established tumour‐related prognostic factors, including pathological stage, nodal status, tumour size, grade, histological subtype and tumour necrosis.^38^ Similarly, recurrence following radical nephroureterectomy for UTUC is influenced by tumour stage, grade, multifocality, tumour location and other patient‐ and treatment‐related factors.^39^ Because the present cohort included heterogeneous malignant pathologies and procedures, and oncological surveillance was not performed according to a standardized study‐specific protocol, recurrence findings were reported descriptively by tumour type. These findings characterize the cohort but should not be interpreted as evidence of oncological equivalence between CLIPS and ENBLOC.

The increasing adoption of robotic‐assisted nephrectomy has introduced new considerations in the selection of renal hilar control techniques. While robotic surgery offers superior three‐dimensional visualization, articulated instrumentation and enhanced dexterity, evidence directly comparing vascular control strategies in this setting remains limited. To our knowledge, this study represents one of the largest comparative cohorts evaluating en bloc stapler ligation and separate clip ligation specifically during robotic‐assisted nephrectomy. Although previous laparoscopic studies have frequently reported shorter operative times with en bloc stapling, we observed no statistically significant difference in operative duration between the two techniques. This discrepancy may reflect the technical advantages of the robotic platform, which facilitate rapid and precise hilar dissection during separate clip ligation, thereby reducing the operative time advantage previously attributed to en bloc stapling in conventional laparoscopy. Consequently, advantages reported in laparoscopic surgery may not necessarily translate to robotic‐assisted procedures, highlighting the importance of evaluating these techniques within the robotic setting rather than extrapolating findings from laparoscopic experience.

From a clinical perspective, both hilar‐control techniques appear technically feasible in contemporary robotic practice, and selection may reasonably be individualized according to hilar anatomy, surgeon experience, institutional practice and device availability or cost. In this cohort, no statistically significant differences were identified in the evaluated primary safety outcomes; however, the observational design, limited number of events and residual confounding preclude conclusions regarding equivalence or superiority. Accordingly, these findings should be considered hypothesis‐generating and support continued individualized technique selection rather than preferential use of either approach.

Cost may also influence the choice of hilar‐control technique, particularly when no clear perioperative advantage of one approach is demonstrated. In direct comparisons of Endo GIA stapling and Hem‐o‐Lok clip ligation during laparoscopic nephrectomy, both Yıkılmaz et al. and Kim et al. reported lower device‐related costs with clip‐based vascular control.^4^, ^40^ Although these findings suggest a potential economic advantage of clips, the available evidence derives from laparoscopic practice, and cost estimates are inherently dependent on institutional procurement, device configuration and local health‐care systems. Accordingly, their applicability to contemporary robotic surgery remains uncertain, and a formal cost analysis was not performed in the present study.

A major strength of this study is its focus on robotic‐assisted nephrectomy, an area in which comparative evidence regarding renal hilar vascular control techniques remains scarce. By evaluating a contemporary cohort that included radical nephrectomy, nephroureterectomy and simple nephrectomy, this study provides clinically relevant evidence that complements the predominantly laparoscopic literature and addresses an important gap in robotic urologic surgery. Additional strengths include the relatively large cohort, inclusion of multiple robotic procedures and exploratory subgroup analyses across three robotic procedures, while recognizing their limited statistical power.

Several limitations warrant consideration. The retrospective, single‐centre, nonrandomized design introduces selection and confounding by indication because the choice of hilar‐control technique was surgeon directed. Despite parsimonious sensitivity analyses, sparse outcome events precluded comprehensive adjustment, and residual confounding related to patient characteristics, procedure type, surgeon, calendar period and unmeasured hilar anatomy or operative complexity cannot be excluded. Pooling three procedures introduced clinical heterogeneity, with the smaller simple nephrectomy and nephroureterectomy cohorts remaining exploratory. Renal function and oncological analyses were descriptive because structured baseline renal function data and recurrence‐event dates suitable for longitudinal or time‐to‐event analyses were unavailable. Although all patients underwent postoperative contrast‐enhanced imaging, surveillance was not AVF‐specific and follow‐up may have been insufficient to detect rare delayed events. Finally, formal cost and resource‐utilization analyses were not performed. These limitations preclude causal or equivalence conclusions.

## 5 CONCLUSION

In this retrospective cohort, en bloc stapler ligation was not associated with a statistically significant difference in perioperative safety compared with separate clip ligation. However, the observational design, residual confounding and limited number of events preclude conclusions regarding equivalence. Technique selection should therefore remain individualized according to hilar anatomy, surgeon experience and procedural context.

## AUTHOR CONTRIBUTIONS

**Ibrahim A. Khalil:** Conceptualization; methodology; data curation; formal analysis; investigation; visualization; writing—original draft; writing—review and editing. **Fatima A. Al‐Jassim:** Data curation; investigation; validation; writing—review and editing. **Yaser M. Ata:** Data curation; investigation; writing—review and editing. **Humam Emad Rajha:** Methodology; formal analysis; validation; writing—review and editing. **Abdulhamed Mansour:** Investigation; data curation; writing—review and editing. **Ibrahim Al‐Emadi:** Data curation; investigation; writing—review and editing. **Khalid Al‐Jalham:** Investigation; supervision; writing—review and editing. **Khalid Al Rumaihi:** Conceptualization; supervision; project administration; methodology; writing—review and editing.

## CONFLICT OF INTEREST STATEMENT

All co‐authors declare no financial or nonfinancial interest in reporting.

## Supporting information


**Table S1.** Histological subtypes among patients with malignant pathology.

## Data Availability

The data that support the findings of this study are not publicly available due to institutional privacy and ethical restrictions. However, the data are available from the corresponding author upon reasonable request and with permission from the relevant institutional review board and participating institution.
