# Supplementary material for: En bloc stapler versus separate clip ligation for renal hilar control during robotic‐assisted nephrectomy: A retrospective comparative cohort study
Source: BJUI Compass. 2026 Oct 16;7(10):e70297. doi: 10.1002/bco2.70297 (PMC13673578; doi:10.1002/bco2.70297)
Supplement: Supplementary file 1 [file BCO2-7-e70297-s001.docx]

**Supplementary Table S1. Histological Subtypes Among Patients With Malignant Pathology**

| **Histological subtype** | **CLIPS (n=45)** | **ENBLOC (n=106)** | **Overall (n=151)** |
| --- | --- | --- | --- |
| Clear-cell RCC | 33 (73.3%) | 90 (84.9%) | 123 (81.5%) |
| Papillary RCC | 10 (22.2%) | 10 (9.4%) | 20 (13.2%) |
| Urothelial carcinoma | 2 (4.4%) | 5 (4.7%) | 7 (4.6%) |
| Other malignant histology | 0 | 1 (0.9%)* | 1 (0.7%) |

Percentages are calculated among patients with malignant pathology within each hilar-control group.* renal leiomyosarcoma.
